# Efficacy of Omega-5 NanoPSO Treatment in the Hippocampus, Through Antioxidant Mechanisms, After an Ischemia/Reperfusion Injury, in Murine Model

**DOI:** 10.3390/antiox13111353

**Published:** 2024-11-05

**Authors:** Irene Guadalupe Aguilar-García, Jonatan Alpirez, José Francisco Muñoz-Valle, Walter Ángel Trujillo-Rangel, David Fernández-Quezada, Sergio Horacio Dueñas-Jiménez, María de la Luz Galvan-Ramírez, María Guadalupe Sánchez-Parada, Ana Elizabeth González-Santiago, Judith Marcela Dueñas-Jiménez, Rolando Castañeda-Arellano

**Affiliations:** 1 Departamento de Neurociencias, Centro Universitario de Ciencias de la Salud, Universidad de Guadalajara, Guadalajara 44100, Jalisco, Mexico; irene.agarcia@academicos.udg.mx (I.G.A.-G.); jonatan.alpirez@alumnos.udg.mx (J.A.); david.fernandez@academicos.udg.mx (D.F.-Q.); sergio.duenas@academicos.udg.mx (S.H.D.-J.); 2 Instituto de Investigación de Ciencias Biomédicas, Centro Universitario de Ciencias de la Salud, Universidad de Guadalajara, Guadalajara 44100, Jalisco, Mexico; drjosefranciscomv@cucs.udg.mx; 3 Laboratorio de Farmacología, Centro de Investigación Multidisciplinario en Salud, Centro Universitario de Tonalá, Universidad de Guadalajara, Tonalá 45425, Jalisco, Mexico; walter.trujillo@academicos.udg.mx; 4 Departamento de Microbiología y Patología, Centro Universitario de Ciencias de la Salud, Universidad de Guadalajara, Guadalajara 44100, Jalisco, Mexico; mlgalvan@cucs.udg.mx; 5 Departamento de Ciencias Biomédicas, División de Ciencias de la Salud, Centro Universitario de Tonalá, Universidad de Guadalajara, Tonalá 45425, Jalisco, Mexico; maria.sparada@academicos.udg.mx (M.G.S.-P.); ana.gonzalez@academicos.udg.mx (A.E.G.-S.); 6 Departamento de Fisiología, Centro Universitario de Ciencias de la Salud, Universidad de Guadalajara, Guadalajara 44100, Jalisco, Mexico; judith.duenas@academicos.udg.mx

**Keywords:** ischemia, omega-5 NanoPSO, neuroprotection, locomotion

## Abstract

Stroke is the third cause of death worldwide and a health problem, and current therapy continues to be very poor. It promotes an alteration associated with excitotoxicity, oxidative stress, and inflammatory processes, exacerbating the damage in the brain. Although cortical areas are the most affected by stroke, the hippocampus can be impacted in the long term through the pathways it connects with these areas, which are associated further with motor alterations; this encourages the search for new therapeutic approaches. Omega-5, being an antioxidant, participates in regulating oxidative stress. A recently designed nanoemulsified compound coupled with pomegranate seed oil (NanoPSO) maintains bioavailability in the body for longer. Omega-5 NanoPSO is more effective in different models of neurodegenerative diseases and metabolic disorders. Therefore, it is important to analyze the effect of omega-5 NanoPSO on ischemic damage through changes in the hippocampus, oxidative mechanisms, and behavioral outcomes. Male Wistar rats were used in five groups; three groups were subjected to an ischemic event through bilateral occlusion of the carotid arteries. An ischemia group received omega-5 NanoPSO after injury, and another group received omega-5 NanoPSO performed two weeks before the ischemic event and three weeks after the surgical process. The control and sham groups did not show changes in the hippocampus and behavior. In the ischemia group, neuronal loss, oxidative stress, and a higher expression of astrocytes were maintained in the hippocampal region, and behavior was modified. In the post and pre-treatment group with omega-5 NanoPSO, we observed reduced damage, glial proliferation, and oxidative stress. It increased neuron survival in the hippocampal region and improved the locomotion. These results highlight its promise for use in clinical settings to treat patients suffering from ischemic brain injury.

## 1. Introduction

Omega-5 NanoPSO, a novel nanoemulsion-based formulation of the antioxidant Punicic acid, has garnered significant interest in ischemic stroke research due to its potential to mitigate the detrimental effects of oxidative stress and neuronal death [1]. Stroke is the leading cause of death and neurological disorders worldwide. The ischemic-type cause accounts for approximately 85% of strokes; it occurs when an intracranial artery is acutely blocked, usually due to an embolism from the heart or arteriosclerotic lesions in proximal arteries [2]. Brain ischemia implicates an alteration of the central nervous system (CNS), causing neuronal death in essential areas; the hippocampus can be impacted in the long term by the pathways it connects with areas susceptible to ischemia, affecting learning, memory, and motor behavior [3]. These cognitive processes are known to decline in the context of neurological injury. Understanding how changes in hippocampal structure impact cognition, including motor function, in the context of ischemic injury is crucial for identifying the critical neural underpinnings of the cognitive process and potential intervention targets. In addition, the blood–brain barrier (BBB) is highly selective and separates circulating blood from the cerebral extracellular fluid in the CNS; its disruption by ischemia alters the homeostatic microenvironment, exacerbating oxidative stress and inflammation associated with impaired behavior [4].

Oxidative stress is a primary driver of neuronal damage and death in the context of ischemic brain injury. Free iron can catalyze the Fenton reaction, producing highly reactive hydroxyl radicals and initiating lipid peroxidation, a key pathological process in CNS disorders. The brain is particularly vulnerable to the effects of reactive oxygen species due to its high oxygen consumption and lipid content and relatively low levels of antioxidant enzymes, such as superoxide dismutase and glutathione peroxidase, compared to other tissues [5]. Therefore, different strategies have been sought to reduce oxidative stress. Omega-5, a conjugated polyunsaturated fatty acid considered one of the most potent natural antioxidants, exhibits various biological properties in reducing free radicals and, consequently, oxidative stress in chronic diseases, immune function enhancement, and lipid metabolism promotion [6,7]. Omega-5 is present only in pomegranate seed oil (PSO) (60–80%), and its lack of toxicity and partial bioavailability have already been established in humans. To increase its bioavailability and activity, it generated water-soluble nano-emulsions of PSO, denominated NanoPSO. Mizrahi et al. administered omega-5 NanoPSO (Granagard^®^) to the Creutzfeldt–Jakob disease model, and the onset and progression of the disease were significantly delayed in the treated animals with NanoPSO [8].

Furthermore, omega-5 NanoPSO treatment prevented age-related cognitive deterioration and mitochondrial oxidative damage in 5XFAD mice, modeling for Alzheimer’s disease [9]. NanoPSO administration to mice before or after TBI application prevents cognitive and behavioral decline, leading to higher SIRT1 and SYP protein post-injury levels [10]. Also, it resulted in decreased body fat mass in the control mice, whereas glucose intolerance, insulin resistance, and hepatic steatosis were improved in control mice and mice fed a high-fat diet [11]; it also reduced the demyelination and oxidation of lipids in the brains of mice induced for experimental autoimmune encephalomyelitis (EAE) [12]. Understanding the mechanisms associated with the antioxidant response of omega-5 NanoPSO (Granagard^®^) in different neurological pathologies could be an effective therapeutic tool. This study aimed to demonstrate brain damage in the hippocampus region caused by ischemic injury, the protective effects of omega-5 NanoPSO, and the ability to restore functions such as motor activity against the brain ischemia model.

## 2. Materials and Methods

### 2.1. Animals and Drug Administration

We used male Wistar rats weighing between 200 and 250 g, who had access to chow and water ad libitum and were placed on a 12 h: 12 h light/dark cycle. The Wistar rats were divided into five different groups: (1) control group, (2) sham group, (3) ischemia group by bilateral common carotid artery occlusion, (4) ischemia+NanoPSO group, and (5) preNanoPSO+ischemia group. In each group, n = 10 was considered. The omega-5 NanoPSO (patent no. 14/523,408) was administered to the two groups of rats in each experiment. The amount of NanoPSO was given directly after ischemic injury at 1.6% oil/mL (10.24 mg equivalent to 2 capsules daily in humans) and administered daily by oral gavage via an intragastric route for three weeks afterward (ischemia+NanoPSO group). While the exact times were two weeks before brain ischemia induction and three weeks after the injury, rats received 1.6% oil/mL administered daily by oral gavage via an intragastric route by adding a self-emulsion formulation (preNanoPSO+ischemia group). All procedures followed the ethical guidelines of the Mexican Official Norm (NOM-062-ZOO-1999), the National Institutes of Health Guide NIH Publication No. 8023 (1996) for the Care and Use of Laboratory Animals and ethical guidelines of University of Guadalajara.

### 2.2. Double Carotid Artery Ligation (DCAL)

We used a model of transient global forebrain ischemia [4]. We permanently ligated the right common carotid artery and transiently clamped the left carotid artery with clamp for 15 min, subsequently removing the clamp and restoring blood circulation to the brain side (event of reperfusion). This model allows us to evaluate both the occluded area and the reperfusion zone in global ischemia, as well as the involvement of critical regions such as the hippocampus. The anterior neck portion was disinfected with a povidone–iodine solution, and the bilateral common carotid arteries were found near the esophagus and trachea. Surgery anesthesia was induced using sevoflurane inhalation at 2% concentration. A thermal mattress monitored and maintained the body temperature at 37–37.5 °C.

### 2.3. Tunnel Walk Recordings

Kinematic records were made one day before and the third and seventh days after the artery occlusion. We identified the videos on day 0 as the control groups (before the injury) and those on the third and seventh days post-injury (3 dpi and 7 dpi) as the lesioned groups. We filmed video recordings of the animals while walking in a tunnel. We used two synchronized cameras that record left and right hindlimbs at 240 fps with a resolution of 1280 × 720 pixels. Post-processing was applied to the resulting videos to remove spherical distortion due to the lenses by estimating a homographic matrix using four points on the image [13]. We selected the animal steps of interest using the instants corresponding to the beginning and end of the step. To obtain the displacement curves of each point of interest (ankle and metatarsus), we manually annotated them on each video frame for each step using custom-made software (Rat System Tracker, version 1.1, Guadalajara, México). We analyzed the values in software developed in our laboratory to assess the kinematic analyses. Each of the steps of the animal captured on the video was analyzed separately.

### 2.4. Dissimilarity Factor Analysis

To assess locomotion kinematic pattern analysis from each point, we generated displacement curves on the horizontal and vertical axes concerning time for each point in the left and right hind limbs during several steps. All curves were normalized according to the stride using a value range from one to 100, employing a spline-based interpolation. We compared their displacement curves to determine changes in the dissimilarity factor (DF) among animal groups. We calculated the difference between them using the Euclidean distance between each of the points of the normalized curve on the horizontal (X) and vertical (Y) axes, as described before [14].

$${\mathrm{DF}}_{\langle a,b\rangle }=\frac{1}{200}\sqrt{\overset{100}{\underset{i=1}{\sum }}{\left(x_{a}\left(i\right)-x_{b}\left(i\right)\right)}^{2}+\overset{100}{\underset{i=1}{\sum }}{\left(y_{a}\left(i\right)-y_{b}\left(i\right)\right)}^{2}}$$

where DF <*a*, *b*> is the squared error between every point of the normalized curves, defined as difference factor (DF); “*x_a_* (*i*) − *x_b_* (*i*)” is the difference between the coordinates in *x* and “*y_a_* (*i*) − *y_b_* (*i*)” in *y* of every point in the graph when comparing two steps (*a* and *b*); and “*i*” is the percent in the step cycle. We compared the curves of every animal in the control and experimental groups, namely the ischemia, ischemia+NanoPSO, and preNanoPSO+ischemia groups. We analyzed the curves of all control animals and the step curves of all experimental animals [14].

### 2.5. Vertical and Horizontal Displacement Analysis

We compared every animal’s curve both on the left side and the right side separately in the control and the ischemia, ischemia+NanoPSO (post 3 dpi or post 7 dpi), and preNanoPSO+ischemia (pre 3 dpi or pre 7 dpi) groups on the third and seventh days post-injury. Every step curve of an animal was compared to the curves of a different control animal sequentially with each displacement curve of all animals in the control group. Thus, we calculated differences in the displacement curves between every animal and compared experimental versus control groups. We graphed all values and statistical hypothesis tests to evaluate group distribution differences. We then determined significant differences between groups for each step phase point for both vertical (Y) and horizontal (X) components. Moreover, we averaged the vector Y components at each end of the normalized displacement curves for each group. We compared this pattern comparison analysis by using a locally designed MATLAB 2023B software. We performed an analysis of displacement 3 and 7 days post-ischemia only, in which we found differences between the left and right sides. This helped us observe differences in motor function associated with the area of carotid occlusion (data shown in Figures S1–S3).

### 2.6. T-Maze Test

Spatial working memory was assessed through the spontaneous alternation and latency T-maze test. The T-maze was mounted 1 m above the floor. All experiments were conducted between 10:00 and 17:00. The T-maze consisted of two arms and one stem (start and lateral goal arms) that were 50 cm long and 10 cm wide. The T-maze task consisted of phase habituation, alternation training, and latency tests (before and after ischemia). The rats were placed in the maze, and cage mates explored it for 5 min. The study was realized once each week for five weeks, modifying the recompense target.

### 2.7. Measurement of BBB Disruption

Vascular permeability was assessed by measuring Evans blue (EB) extravasation with quantitative fluorescence. Briefly, the rats received 1.2 mL/kg of Evans blue solution (Sigma E2129, Darmstadt, Germany) in saline by intraperitoneal (IP) injection 30 min after focal cerebral ischemia injury. To evaluate brain edema, the animals were decapitated 5 days after ischemia. The brains were homogenized to extract the Evans blue, and to precipitate protein, 1 mL of 70% trichloroacetic acid was added and mixed by vortex for 1 min. The samples were placed at 4 °C overnight and centrifuged for 30 min at 1000× *g* at 4 °C. The 100 µL of total supernatants was measured at 620 nm using a spectrophotometer (BioTek Synergic HT, Winooski, VT, USA). The dye concentration was calculated as the absorbance ratio relative to the tissue amount. Extravasated EB dye was expressed as µM concentration of brain tissue.

### 2.8. Cresyl Violet Staining

Three weeks after the brain ischemia injury, the animals were anesthetized intraperitoneally with pentobarbital at 160 mg/kg of body weight. We sacrificed the animals by intracardiac perfusion using a saline solution (0.9%) and 4% paraformaldehyde. Brains were removed and placed in the same fixed solution at 4 °C. We made 20 μm of vibratome sections for the brain coronal (Leica, VT1000s, Wetzlar, Germany). We divided it into 20 serial sections for Nissl staining in each group. The slices were put in 15 min in 100% xylene, then 10 min in 100% ethanol. They were rehydrated through alcohol 100% for 3 min each, washed in tap water, stained in 0.1% Cresyl violet (Sigma C5042, Darmstadt, Germany) for 4–15 min, and washed in 70% ethanol (this method removed the stains). Then, they were dehydrated through 3 min changes of absolute ethanol and cleared in xylene two times. We used Entellan for mounting sections of Nissl staining and observed them under a microscope (Carl-Zeiss Oberkochen, Germany) at 10X and 40X. We were interested in the hippocampus area, primarily the CA1 region, and counted cells using a 40X objective, considering four fields of the ipsilateral hemisphere to permanent occlusion. 

### 2.9. Hematoxylin and Eosin Staining

We evaluated another area of the hippocampus that may be compromised, such as the CA3, and whether it could show different changes compared to those analyzed in CA1 using the Nissl technique. We used hematoxylin and eosin (H&E) staining. Coronal sections of 30 µm thickness were cut using a vibratome, and ten nerve tissue sections were randomly selected. Sections were immersed in hematoxylin solution for 5–10 min, rinsed in running tap water for 5 min, and differentiated in 0.3% acid alcohol (1% hydrochloric acid in 70% ethanol) for a few seconds until light blue. This was followed by rinsing in running tap water for 5 min. Sections were then stained in eosin solution for 1–3 min and briefly rinsed in distilled water to remove excess stains. A drop of the mounting medium was applied to each section. Positive H&E staining was localized and photographed. Briefly, H&E-positive neurons were imaged using a Leica DMi8 microscope (Leica Microsystems Inc., Bannockburn, IL, USA) with a 20× objective to obtain two photographs of the CA3 region in 8-bit grayscale. Each picture represented a microscopic field in which the dimensions of the CA3 region were covered entirely. Images were acquired with a DFC 7000T Leica camera at a resolution of 1296 × 972 pixels in the *x*- and *y*-axis (0.22 px/μm).

### 2.10. Immunofluorescence to GFAP+Cells

We evaluated the astrogliosis caused by the brain ischemia injury using GFAP-immunofluorescence three weeks after reperfusion. Free-floating 20 μm sections underwent incubation at room temperature for 30 min in PBS 1x/Triton X-100 0.2%. Next, sections were incubated for 1 h in PBS 1x/BSA 1%, then with GFAP antibody (1: 500, Invitrogen Waltham, MA, USA) overnight and subsequently incubated with the secondary antibody (FITC anti-rabbit IgG, Jackson ImmunoResearch, West Grove, PA, USA) for 2 h. Sections were analyzed with a laser confocal scanning microscope (Olympus, FV300, Tokyo, Japan) with a 40 oil-immersion objective.

### 2.11. Measurement of Total Antioxidant Capacity and Lipoperoxidation Levels

We obtained a blood sample from the rats, and the serum was separated. We prepared a series of standard solutions using the Trolox standard by diluting it with the assay buffer (CS0790 Sigma, Darmstadt, Germany). These standards were used to create a calibration curve. Then, the samples were diluted with the assay buffer. In a 96-well microplate, we added 100 µL of each standard and sample to separate wells, along with 100 µL of the Cu^2+^ working solution to each well to initiate the reaction. Also, the plate was incubated at room temperature for 30 min. The antioxidants in the sample reduce Cu^2+^ to Cu^1+^, which reacts with the chromogenic reagent. We added 100 µL of the chromogenic detection reagent to each well; the measure absorbance was found to be 570 nm using a microplate reader. The intensity of the color was proportional to the sample’s antioxidant capacity. We mixed the serum in ice-cold PBS for lipid peroxidation to avoid further lipid peroxidation. BHT was added to the samples to prevent further lipid peroxidation during the assay and after centrifuging at 10,000× *g* for 5–10 min at 4 °C. We mixed equal volumes of the thiobarbituric acid (TBA) and phosphoric acid solutions in the kit to prepare a TBA reagent mixture (AB233471 Abcam, Cambrige, UK). We added a measured volume of 100 µL of the sample or standard to each test tube and the TBA reagent mixture to each tube, with 2 mL per 100 µL of the sample. We incubated the samples and standards at 95 °C in a water bath or heating block for 60 min. This heat treatment allowed TBA to react with malondialdehyde (MDA), forming a pink chromogen. The absorbance was measured at 532 nm using a spectrophotometer. The absorbance is directly proportional to the MDA concentration.

### 2.12. Measurement of Superoxide Dismutase Total

For the evaluation of the superoxide dismutase (SOD) enzyme (CS0009 Sigma, Darmstadt, Germany), EDTA in sodium cyanide and nitro blue tetrazolium (NBT) were added to a specific volume of serum. After mixing, it was placed at 37 °C. Then, riboflavin and methionine were added to the samples, increasing the volume to 3 mL. Then, the sample was placed at environmental temperature and light for 10 min. Then, the absorption of samples and control (distilled water) was read at 560 nm for 5 min. Enzyme activity was reported based on units per mg of protein.

### 2.13. Nitrite/Nitrate Concentration

Nitric oxide production in the serum was determined by tissue accumulation of nitrite and nitrate using a colorimetric assay (23479 Sigma, Darmstadt, Germany). Nitrite/nitrate (N/N) concentration was measured by Griess reaction in serum samples by adding 100 μL of Griess reagent 0.1% (*w*/*v*) naphthylethylendiamide dihydrochloride in H_2_O and 1% (*w*/*v*) sulphanilamide in 5% (*v*/*v*) concentrated H_3_PO_4_, vol. (1:1) to the 100 μL sample. After one hour of incubation at room temperature, absorbance was recorded in a spectrophotometer at 550 nm. The results were expressed as nmol of N/N concentration per milligram of protein.

### 2.14. Experimental Design and Statistical Analysis

For multiple comparisons, we used a one-way ANOVA test followed by post hoc *t*-tests with Bonferroni corrections; values of * *p* < 0.05, ** *p* < 0.01, *** *p* < 0.001, **** *p* < 0.0001 was considered statistically significant. To compare the means of the two groups, we used a student’s *t*-test; values of * *p* < 0.05, ** *p* < 0.01, *** *p* < 0.001 were considered statistically significant. We used a non-parametric of the Kruskal–Wallis test followed by Dunn’s. All results were expressed as means ± S.E.M. Statistical analyses were performed using the GraphPad Prism 10.4 Software (La Jolla, CA, USA) and MATLAB 2023B software (Natick, MA, USA).

## 3. Results

### 3.1. The Changes in Displacement on the Third and Seventh Days Post-Brain Ischemia Injury with Omega-5 Nanopso Treatment

**Figure 1:**
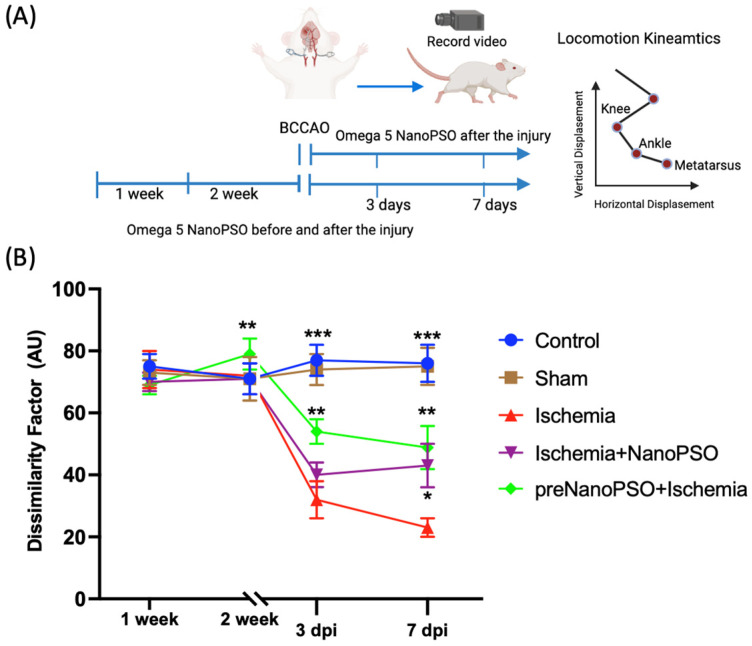
Analysis in the displacement of rats after ischemia injury and omega-5 NanoPSO treatment. (**A**) Schematic representative about the timeline of omega-5 NanoPSO treatment before and after induction of ischemia/reperfusion and analyzed by displacement curves. (**B**) Dissimilarity factor (DF) in the left and right hindlimb. The lines in the graphic illustrate control, sham, ischemia, ischemia+NanoPSO, and preNanoPSO+ischemia groups. They show mean ± SEM values. We calculated statistical differences between groups using the ANOVA test. *p* ≤ 0.05 (*), *p* ≤ 0.01 (**), *p* ≤ 0.001 (***). The vertical and horizontal displacement was averaged and compared between control versus lesioned and treated rats.

The displacement was graphical, analyzing the dissimilarity factor (DF). Figure 1A describes the timeline of treatment with omega-5 NanoPSO in the two study groups and the analysis of vertical and horizontal displacement to assess locomotion kinematic patterns. In Figure 1B, five study groups were analyzed. The control and sham groups did not show differences in DF, while the ischemia group observed a difference of 63% in DF concerning the control group (*p* < 0.001) and differed by 35% from the preNanoPSO+ischemia (*p* < 0.01) 3 days post-injury (dpi); the ischemia+NanoPSO group did not differ significantly. On the seventh day post-injury, the ischemia group showed a significant difference of 65% compared to the control group (*p* < 0.001) and of 37% in the preNanoPSO+ischemia group (*p* < 0.01). There was also a difference in the ischemia+NanoPSO group (*p* < 0.05) when analyzing the DF in this study. Interestingly, the preNanoPSO+ischemia group was administered omega-5 NanoPSO 2 weeks before the injury, and the analysis of displacement one day before showed a difference of 13% concerning all groups (*p* < 0.05).

### 3.2. The Post-Treatment with Omega-5 Nanopso Modified Vertical (VD) and Horizontal Displacement (HD) in the Metatarsus, Ankle, and Knee After Ischemia/Reperfusion

**Figure 2:**
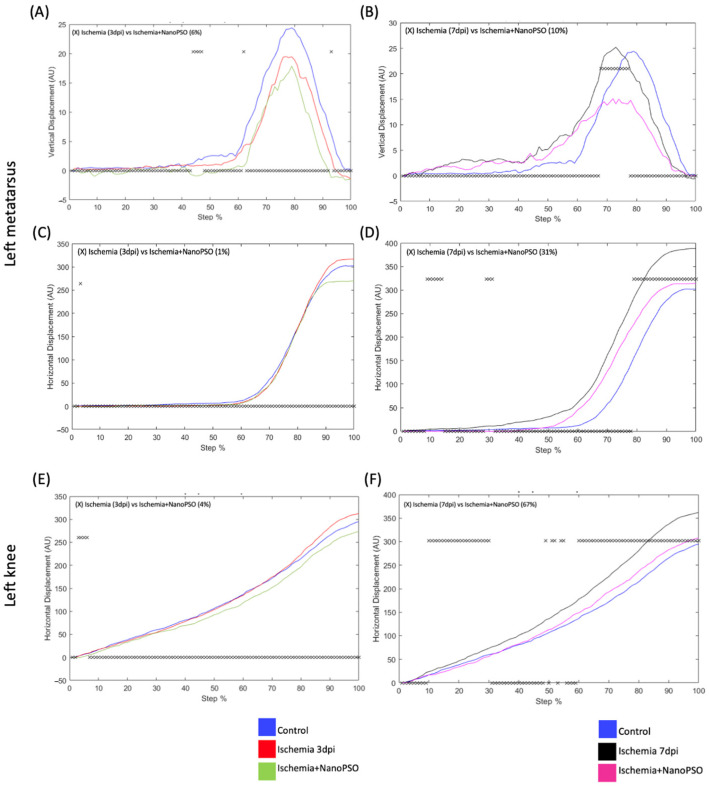
Analysis of vertical (VD) and horizontal (HD) displacement in left metatarsus and knee treated with omega-5 NanoPSO after ischemia injury. (**A**,**B**) Analysis of VD in the left metatarsus. All analyses were performed to compare with ischemia 3 and 7 days post-injury versus ischemia+NanoPSO. (**C**,**D**) Analysis of HD in the left metatarsus. All analyses were performed to compare with ischemia in 3- and 7-days post-injury versus ischemia+NanoPSO. (**E**,**F**) Analysis of HD in the left knee. All analyses were performed to compare with ischemia 3 and 7 days post-injury versus ischemia+NanoPSO. The step cycle was divided into 100 bins (cycle percentage). The cross above Zero indicates bins with a significant statistical difference. The blue line illustrates the control, and the red and black lines illustrate the ischemia at 3 and 7 days, respectively. The green and pink lines represent the ischemia+NanoPSO (pre 3 and 7 dpi) groups. Significant statistical differences (*p* ≤ 0.05) were obtained using Student’s *t*-test. The symbol (x) above (0) represents each point where there were significant differences between the evaluated groups.

To be more specific, at what point there were changes in the kinematics, we analyzed the displacement of vertical and horizontal steps in the left and right joints. The curve data were analyzed compared to the control group (blue line). However, we did not find significant differences on the right metatarsus, ankle, and knee as evident as on the left (data shown in Table S1). On the third day post-injury (red line), the VD differed by 6% of the step cycle in the left metatarsus compared to the ischemia+NanoPSO group (green line) (Figure 2A), but on the seventh day post-injury (black line), the VD differed by 10% of the step cycle in the left metatarsus compared to the ischemia+NanoPSO group (pink line) (Figure 2B). HD in the 3-day post-injury differed by 1% in the left metatarsus concerning ischemia+NanoPSO (Figure 2C). The HD on the seventh day post-injury differed by 31% in the left metatarsus compared to the ischemia+NanoPSO group (Figure 2D). The VD and HD of the left ankle did not change in the injury group versus the ischemia+NanoPSO group. On the third day post-injury, in the left knee, we did not find differences in the VD, whereas the HD differed by 4% compared to the ischemia+NanoPSO group (Figure 2E). The HD on the seventh day post-injury differed by 67% in the left metatarsus compared to the ischemia+NanoPSO group (Figure 2F).

### 3.3. The Pre-Treatment with Omega-5 Nanopso Modified VD and HD in the Metatarsus, Ankle, and Knee After Ischemia/Reperfusion

**Figure 3:**
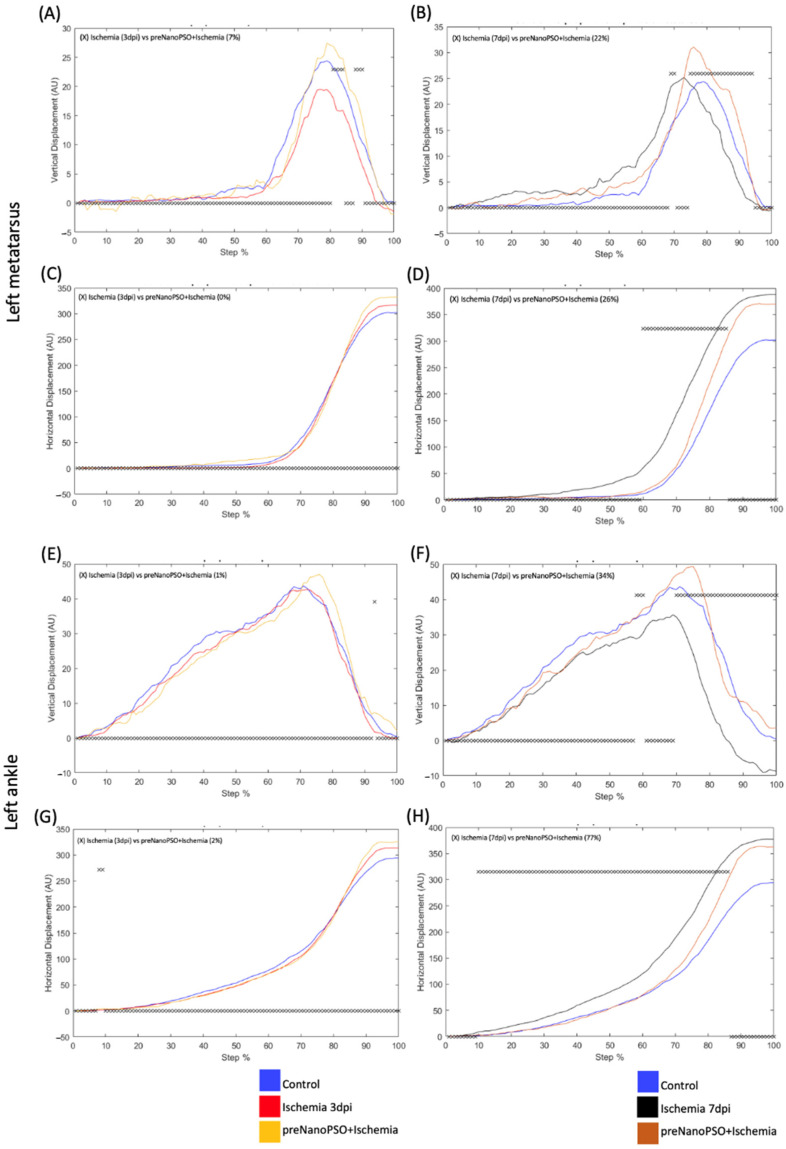
Analysis of vertical (VD) and Horizontal (HD) displacement in left metatarsus and ankle treated previously with omega-5 NanoPSO and after ischemia injury. (**A**,**B**) Analysis of VD in the left metatarsus. All analyses were performed to compare with ischemia 3 and 7 days post-injury versus preNanoPSO+ischemia. (**C**,**D**) Analysis of HD in the left metatarsus. All analyses were performed to compare with ischemia 3 and 7 days post-injury versus preNanoPSO+ischemia. (**E**,**F**) Analysis of VD in the left ankle. All analyses were performed to compare with ischemia 3 and 7 days post-injury versus preNanoPSO+ischemia. (**G**,**H**) Analysis of HD in the left ankle. All analyses were performed to compare with ischemia 3 and 7 days post-injury versus preNanoPSO+ischemia. The step cycle was divided into 100 bins (cycle percentage). The cross above Zero indicates bins with a significant statistical difference. The blue line illustrates the control, and the red and black lines illustrate the ischemia at 3 and 7 days, respectively. The yellow and brown lines represent the preNanoPSO+ischemia (Pre 3 and 7 dpi) groups. Significant statistical differences (*p* ≤ 0.05) were obtained using Student’s *t*-test.

**Figure 4:**
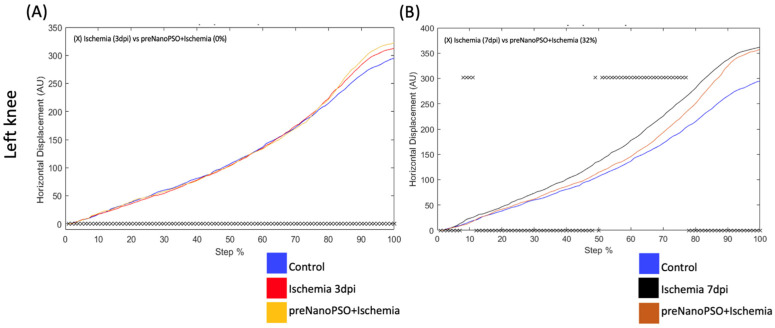
Analysis of horizontal displacement (HD) in left knee treated previously with omega-5 NanoPSO and after ischemia injury. (**A**) Analysis of HD in the left knee was performed to compare with ischemia 3 days post-injury versus preNanoPSO+ischemia. The step cycle was divided into 100 bins (cycle percentage). The asterisk above zero indicates bins with a significant statistical difference. (**B**) Analysis of HD in the left knee compared with ischemia 7 days post-injury versus preNanoPSO+ischemia. The step cycle was divided into 100 bins (cycle percentage). The cross above zero indicates bins with a significant statistical difference. The blue line illustrates the control, and the red and black lines illustrate the ischemia at 3 and 7 days, respectively. The yellow and brown lines represent the preNanoPSO+ischemia (pre 3 and 7 dpi) groups. Significant statistical differences (*p* ≤ 0.05) were obtained using Student’s *t*-test.

The pre-treatment of omega-5 NanoPSO helped us see if prior administration can maintain or improve motor functions in brain injury. In the ischemia group, 3 days post-injury (red line), the VD differed by 7% of the step cycle in the left metatarsus compared to the preNanoPSO+ischemia group (yellow line) (Figure 3A). The VD on the seventh day post-injury (black line) differed by 22% in the left metatarsus compared to the preNanoPSO+ischemia group (brown line) (Figure 3B). The HD on the third day post-injury did not differ concerning the preNanoPSO+ischemia group (Figure 3C). On the seventh day post-injury, the HD differed by 26% in the step cycle in the left metatarsus compared to the preNanoPSO+ischemia group (Figure 3D). On the seventh day post-injury, the VD differed by 34% of the step cycle in the left metatarsus compared to the preNanoPSO+ischemia group (Figure 3E). In this case, the HD was different in the right ankle when compared 3 days post-injury to the preNanoPSO+ischemia group by 2% (Figure 3F). On the seventh day post-injury in the ischemia group, the HD differed by 77% of the step cycle in the left ankle compared to the preNanoPSO+ischemia group (Figure 3G). The left knee did not show a different VD compared to the third and seventh days post-injury in the preNanoPSO+ischemia group. On the third day post-injury in the left knee, the HD did not differ from that of the preNanoPSO+ischemia group (Figure 4A). On the seventh day post-injury, the HD differed by 32% of the step cycle in the left knee compared to the preNanoPSO+ischemia group (Figure 4B).

### 3.4. Omega-5 NanoPSO Treatment in the Ischemia/Reperfusion Model Modified Spontaneous Alternation and Latency in the T-Maze Test

**Figure 5:**
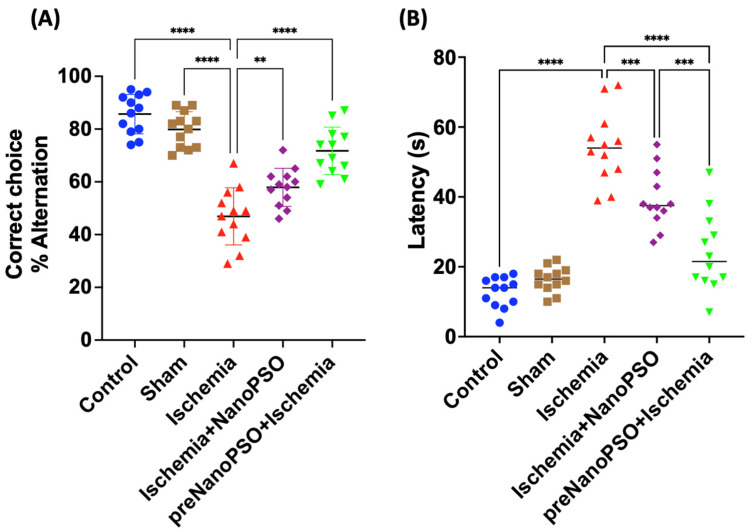
Correct Choice and latency analysis after brain ischemia and with the treatment of omega-5 NanoPSO. (**A**) Percent of correct choice as a function of the test day, as measured in the T-maze apparatus. (**B**) Latency to choose the arm in the second trial, meantime (sec) the rats spent out of the area. Fisher’s exact test was used for categorical data analysis (*n* = 10/group). Comparisons among groups were performed with the non-parametric Kruskal–Wallis test, followed by Dunn’s post hoc test. The ** *p* < 0.01, *** *p* < 0.001, **** *p* < 0.0001 values were considered statistically significant.

We analyzed the alternation and latency associated with memory in rats. The control and sham groups showed a quick choice capacity with few errors of 16% and 19%, respectively. In contrast, the ischemia group showed a spatial working memory reduction of 48% compared to the control group (*p* < 0.0001). In addition, the ischemia+NanoPSO group showed a 12% improvement compared to the ischemia group (*p* < 0.01). The preNanoPSO+ischemia group showed a more significant improvement of 29% in contrast with the ischemia group (*p* < 0.0001) (Figure 5A). We perform the analysis concerning latency in seconds. The control and sham groups showed a fast travel time, and no differences existed between them. In the ischemia group, the latency (54.50 ± 12.41) was more significant than the control group. In addition, the ischemia+NanoPSO group reduced the latency (14.92 ± 4.45) compared to the ischemia group. The preNanoPSO+ischemia group decreased the latency (30.17 ± 9.01) compared to the ischemia group (*p* < 0.0001) and (15.25 ± 4.5) compared to the ischemia+NanoPSO group (*p* < 0.001) (Figure 5B).

### 3.5. The Omega-5 NanoPSO Decreases Edema in the Brain After Brain Ischemic Injury

**Figure 6:**
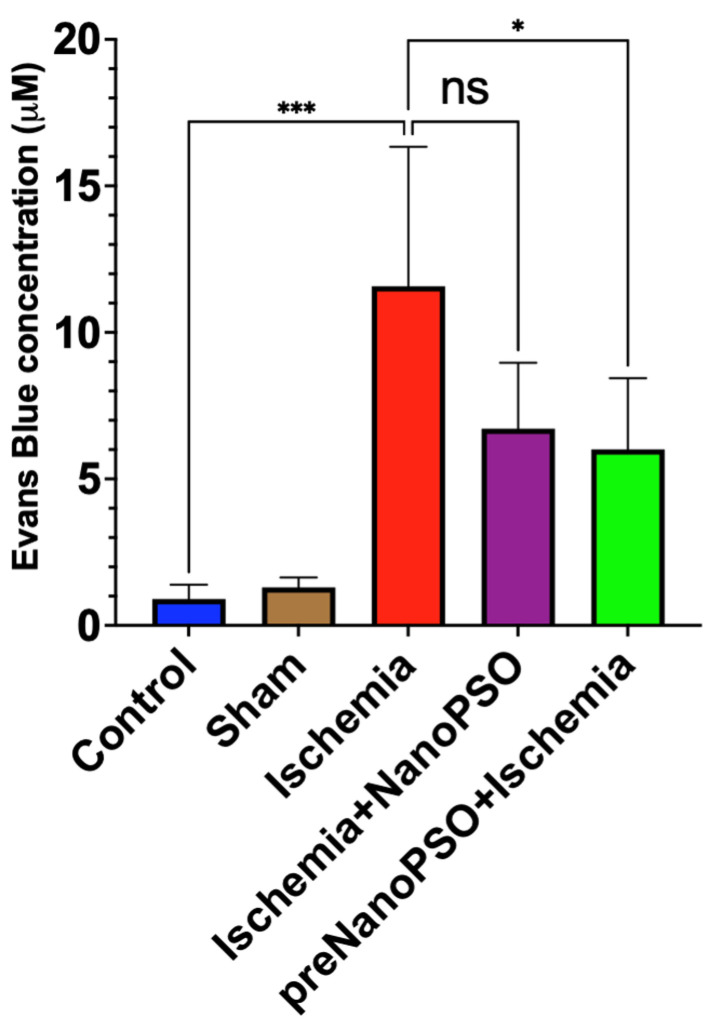
Extravasation analysis by Evans blue after brain ischemia injury and omega-5 NanoPSO treatment. Analysis of Evans blue extravasation (μM) 5 days after ischemic injury in rats. Treatment with omega-5 NanoPSO significantly reduced Evans blue extravasation in the brain. We evaluated the control, sham, ischemia, ischemia+NanoPSO, and preNanoPSO+ischemia groups (*n* = 10/group). Values are mean ± SEM. We calculated statistical differences between groups using the ANOVA test. *p* ≤ 0.05 (*), *p* ≤ 0.001 (***).

In this study, we aimed to evaluate the impact of omega-5 NanoPSO on post-ischemic edema using the Evans blue dye extravasation technique, which provides a quantitative measure of blood–brain barrier permeability. In the ischemia group, we found that extravasation to the brain was marked by Evans blue. Interestingly, the groups that received ischemia+NanoPSO showed a downward trend, but it was insignificant since they received one week of treatment; however, the preNanoPSO+ischemia group demonstrated decreased Evans blue extravasation in brain parenchyma of 44% concerning the ischemic group (*p* < 0.01). The control and sham groups did not show any significance, but the ischemic group was different (*p* < 0.001) (Figure 6).

### 3.6. The Omega-5 NanoPSO Decreases Histological Damage in CA1 and CA3 After an Ischemic Insult

**Figure 7:**
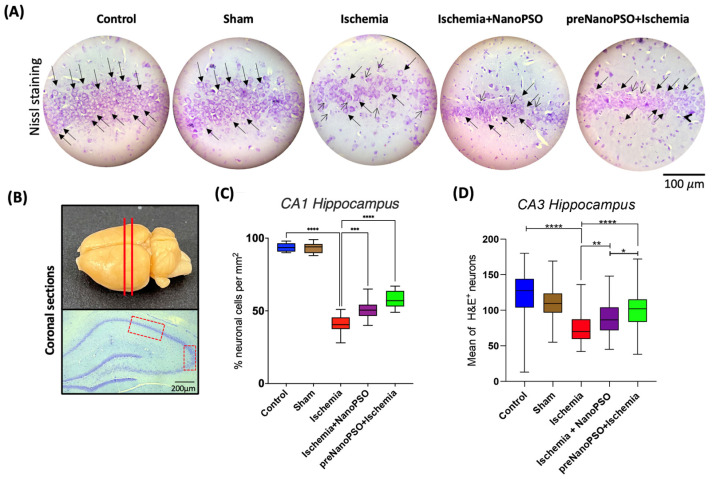
Changes in the survival and death neuronal of the CA1 and CA3 of the hippocampus after brain ischemia and with the treatment of omega-5 NanoPSO. (**A**) Representative photography of the CA1 area showing the neuronal cells with nuclei defined (black arrows) and pyknotic nuclei (arrow in the beak) in the control, sham, ischemia, ischemia+NanoPSO, and preNanoPSO+ischemia groups. (**B**) The brain section was cut with coronal slices (red lines), and the hippocampus region showed the CA1 and CA3 areas (red box dashed lines). (**C**) Neuronal cell percentage in the CA1 area counting intact nuclei of five study groups (*n* = 10/group). Values are mean ± SEM. We calculated statistical differences between groups using the ANOVA test. *p* ≤ 0.001 (***), *p* ≤ 0.0001 (****). (**D**) Mean of H&E neurons in the CA3 area. Values are mean ± SEM. We calculated statistical differences between groups using the ANOVA test. *p* ≤ 0.05 (*), *p* ≤ 0.01 (**), *p* ≤ 0.0001 (****). Scale bar 100 µm and 200 µm.

Representative photomicrographs of histological sections in the hippocampus of the five study groups show neurons of average size using the Nissl staining. Black arrows show the defined nuclei of pyramidal cells in CA1, and the arrow in the beak shows retraction nuclei in the CA1 area (Figure 7A). The sections were cut corresponding to the hippocampus region in the rat brain (up figure), and the histological sections of the hippocampus were visualized for the red-dotted CA1 and CA3 areas (Figure 7B). The analysis with Nissl staining for the percentage of neuron cells demonstrated that 60% of cell deaths were in the ischemic group versus the control group (*p* < 0.0001). Compared with the omega-5 NanoPSO groups, the ischemia+NanoPSO group’s neuronal death decreased by 11% in the CA1 area compared to the ischemia group and 19% compared to the preNanoPSO+ischemia group, which is statistically significant (Figure 7C). Another analysis was performed with H&E in the CA3 area because it is susceptible. We found a similar pattern in cell counting, with 53% of neuronal loss in the ischemic group versus the control group (*p* < 0.0001). The differences in the ischemia+NanoPSO and preNanoPSO+ischemia group scompared to the ischemic group were 13% and 17%, respectively (Figure 7D).

### 3.7. The Omega-5 NanoPSO Decreases Astrogliosis in the Hippocampus After an Ischemic Injury

**Figure 8:**
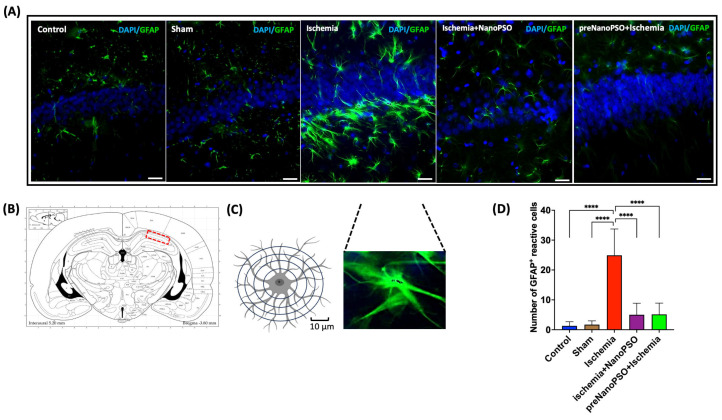
Astrogliosis in brain ischemia and treated with omega-5 NanoPSO (**A**) Microphotograph showing the merge of GFAP+cells (green), and cell nuclei stained with DAPI (blue) in the CA1 hippocampal area of all groups: the control, sham, ischemia, ischemia+NanoPSO, and preNanoPSO+ischemia (*n* = 10/group), scale bar = 50 µm. (**B**) Paxinos atlas shows Bregma coordinates −3.80 mm and the red rectangle with a dotted line. (**C**) The presence of elongated processes in the astrocyte. Representative photo of an elongated process. Scale bar 10 µm. (**D**) The number of GFAP+cells in the CA1 area was markedly higher in the ischemia group; all groups were accounted for. Values are mean ± SEM. We calculated statistical differences between groups using the ANOVA test. *p* ≤ 0.0001 (****).

Astrogliosis is a phenomenon that occurs after ischemia. Photographic representative images of GFAP+cells were taken under different conditions; the nuclei were marked with DAPI (blue) and GFAP+cells (green) (Figure 8A). According to the coordinates of Paxino, we show the selected area with a red dotted line corresponding to the CA1 region. (Figure 8B). Reactive glia is characterized by the presence of elongated processes that extend throughout the area around the nucleus in the astrocyte. We took a representative photo of an elongated process (Figure 8C). We counted GFAP+cells; the control and sham groups showed few GFAP+reactive cells, whereas, in the ischemia group, the number was 25 cells by field on average; in just the ischemia+NanoPSO or preNanoPSO+ischemia groups, the number of GFAP+reactive cells decreased by 75% (*p* < 0.0001) (Figure 8D).

### 3.8. The Effect of Omega-5 NanoPSO Treatment on Oxidative Stress Markers in an Ischemia/Reperfusion Model

**Figure 9:**
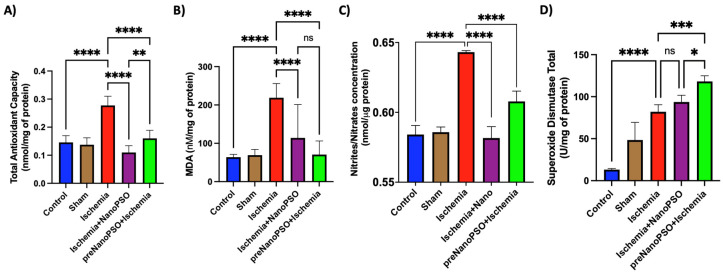
Oxidative stress analyzed by TAC, MDA, nitrites/nitrates, and SOD total after ischemia and omega-5 NanoPSO. (**A**) Quantification of total antioxidant capacity (TAC), (**B**) malondialdehyde (MDA), (**C**) nitric oxide metabolites as nitrites/nitrates, and (**D**) superoxide dismutase total in the control, sham, ischemia, ischemia+NanoPSO, and preNanoPSO+ischemia (*n* = 10/group). Values are mean ± SEM. We calculated statistical differences between groups using the ANOVA test. *p* ≤ 0.05 (*), *p* ≤ 0.01 (**), *p* ≤ 0.001 (***), *p* ≤ 0.0001 (****).

We evaluated oxidative stress levels using TAC, MDA nitrites/nitrates, and SOD after brain ischemia. The TAC showed levels significantly higher in the ischemia group than in all other groups (*p* < 0.0001); interestingly, the post and preNanoPSO+ischemia groups showed a reduction in TAC (*p* < 0.0001); in turn, the ischemia+NanoPSO group observed a significant decrease in TAC (*p* < 0.01) compared to the preNanoPSO+ischemia group (Figure 9A). For the analysis of lipoperoxidation, we measured MDA levels, which were significantly higher in the ischemia group (*p* < 0.0001) than in the control and sham groups. Comparing the ischemia+NanoPSO and preNanoPSO+ischemia groups, the same trend was observed, with the MDA concentration diminishing (*p* < 0.0001) in both groups (Figure 9B). To measure nitric oxide, we evaluated its metabolites, such as nitrates/nitrates. It was observed that the ischemia group had a more significant amount of these compounds metabolized from nitric oxide compared to the control and sham groups (*p* < 0.0001). Interestingly, when we used omega-5 NanoPSO in the ischemia+NanoPSO or preNanoPSO+ischemia groups, the ratio of metabolites was reduced (*p* < 0.0001) (Figure 9C). Another evaluation was done to measure the total of superoxide dismutase. Interestingly, in the ischemia group, the concentration was statistically significantly higher than in the control group (*p* < 0.0001). The sham group only showed a trend that was not significant. In this regard, no significant differences were shown when evaluating the ischemia+NanoPSO group compared to the ischemia group. Notoriously, the preNanoPSO+ischemia group showed an increase in the superoxide dismutase total (*p* < 0.001) compared to the ischemia group. Still, it showed a rise in the post-treatment group (*p* < 0.05) (Figure 9D).

## 4. Discussion

Ischemic brain injury is a devastating condition that can lead to significant morbidity and mortality. The primary method to reduce or prevent neurologic damage in patients suffering from brain ischemia is to restore blood flow to the ischemic tissue [15]. However, this paradoxical reperfusion can exacerbate neurocognitive deficits by producing excessive reactive oxygen species and damaging cellular components. Mitochondria play a critical role in reperfusion injury, as they are the primary source of reactive oxygen species and initiate cell death pathways [15]. Strategies to mitigate the detrimental effects of reperfusion, such as using antioxidants, have shown promise in animal models of ischemic brain injury. One potential therapeutic candidate is omega-5 NanoPSO encapsulated in a nanoparticle delivery system. The present study aimed to investigate the effects of omega-5 NanoPSO on functional outcomes in an animal model of cerebral ischemia-reperfusion injury. Here, we report that compared to the ischemia-only group, the groups that received omega-5 NanoPSO improved their displacement after 3 and 7 days. This finding suggests that omega-5 NanoPSO may protect against the detrimental consequences of ischemia-reperfusion injury. The mechanisms by which omega-5 NanoPSO exerts its neuroprotective effects are not fully understood but may involve its potent antioxidant properties and ability to modulate mitochondrial function.

Further investigation is warranted to elucidate the underlying molecular pathways and to assess the clinical potential of this novel therapeutic approach. Existing rehabilitation strategies have often focused on behavioral training, with limited direct targeting of the underlying neurological mechanism [16]. However, recent advancements in neuroscience and rehabilitation technology have opened up new avenues for improving motor recovery in stroke patients.

We demonstrated that omega-5 NanoPSO influences changes in vertical and horizontal displacement patterns after ischemic injury. This treatment targets specific neurological pathways and can enhance motor function in stroke patients. The existing literature suggests that omega treatment may positively impact motor recovery in stroke patients. Specifically, studies have reported improvements in neuromuscular activation during reach tasks, increased plasticity in the sensorimotor cortex, and enhanced recovery of motor deficits through various rehabilitation interventions [17,18,19]. These findings indicate that omega-5 NanoPSO treatment could be a valuable addition to comprehensive stroke rehabilitation programs. As the understanding of the mechanisms underlying motor recovery after stroke continues to evolve, the integration of targeted interventions like omega-5 NanoPSO treatment with traditional rehabilitation strategies may lead to more effective and personalized care for stroke patients [16,18,20,21] the ischemic brain can lead to a breakdown of the blood–brain barrier, exacerbating neurological damage and impairing recovery [22]. One potential therapeutic approach is using marine algae-derived compounds, such as omega-5 NanoPSO, which have demonstrated neuroprotective effects. Oxidative stress is a key driver of blood–brain barrier disruption following ischemic injury. This allows for the influx of harmful molecules and inflammatory cells, further exacerbating brain injury [23]. Restoring the integrity of the blood–brain barrier is crucial for limiting further damage and promoting neurological recovery.

Our findings indicate that the omega-5 NanoPSO effectively reduced the extravasation of Evans blue dye, a marker of blood–brain barrier integrity, in the ischemic region of the brain. This suggests that the omega-5 NanoPSO restored the blood–brain barrier function and limited the leakage of potentially harmful substances into the brain tissue. The neuroprotective effects of the omega-5 NanoPSO can be attributed to its unique properties as a nanoparticle-based drug delivery system. Nanoparticles can cross the blood–brain barrier and deliver therapeutic agents directly to the site of injury [24,25,26,27].

On the other hand, Ischemic brain injury is a devastating condition that can lead to neuronal death and impaired brain function. Several strategies have been investigated to mitigate this oxidative stress and promote neuronal survival, including antioxidant compounds [23,28]. This study investigated the neuroprotective effects of omega-5 NanoPSO in an ischemic brain injury induced by 15 min of occlusion. Our findings indicate that both pre-treatment and post-treatment with omega-5 NanoPSO significantly reduced the number of neuronal deaths compared to the ischemic group. This highlights the compound’s ability to use omega-3 and other antioxidants to mitigate the detrimental effects of ischemic insult on the brain [29,30]. Furthermore, several kinds of omegas have been shown to modulate key intracellular signaling pathways, such as activating protective transcription factors and downregulating pro-apoptotic mediators [27]. These pleiotropic effects of omega-5 NanoPSO would also underscore its potential as a comprehensive neuroprotective strategy for managing ischemic brain injury.

The inflammatory process is characterized by the activation and proliferation of astrocytes, which can exacerbate tissue damage and impair neurological function after brain ischemic injury [31]. A well-known Omega with anti-inflammatory properties is omega 5 [31], but novel therapeutic strategies to mitigate brain ischemia have been an intense focus. One promising candidate that has garnered significant attention is the omega-5 NanoPSO. In our study, the omega-5 NanoPSO decreases astrogliosis in both groups, which means an essential role of this omega. Furthermore, the compound’s anti-inflammatory actions, mediated through the modulation of proinflammatory signaling cascades, have been demonstrated to confer additional neuroprotective benefits. Oxidative stress, an imbalance between pro-oxidants and antioxidants, is a well-established factor in the pathogenesis of various neurodegenerative diseases [32]. The central nervous system is particularly vulnerable to oxidative stress due to its high oxygen demand, lipid content, and weak antioxidant defenses [23].

We investigated the potential regulatory effects of omega-5 NanoPSO on key oxidative stress markers, including lipoperoxidation, total antioxidant capacity, nitrites and nitrates, and superoxide dismutase. Lipoperoxidation, a well-known consequence of oxidative stress, can form reactive aldehydes and damage cellular membranes. Omega-5 NanoPSO significantly reduced lipoperoxidation levels in brain ischemia injury, suggesting its ability to mitigate oxidative damage to lipids since lipid peroxidation can disrupt cell membranes, impair cellular functions, and ultimately result in cell death [33]. Research has shown that the total antioxidant capacity can play a crucial role in the outcome of ischemic stroke patients [34]. A study of malignant middle cerebral artery infarction patients found an association between total antioxidant capacity levels and mortality and further identified a relationship between lipid peroxidation state and mortality after severe ischemic stroke [35]. Interestingly, in our brain ischemia model, we found that when rats received omega-5 NanoPSO, the total antioxidant capacity decreased significantly, suggesting a potential protective role of antioxidants. The increased prevalence of modifiable risk factors highlights the need for a better understanding of the role of antioxidant status in this population. During ischemia, nitric oxide availability is decreased, leading to endothelial dysfunction and increased oxidative stress [36]. As reperfusion occurs, the sudden restoration of oxygen can induce further damage by forming reactive nitrogen species, such as peroxynitrite, which can cause lipid peroxidation, protein oxidation, and DNA damage [37]. Omega-5 NanoPSO has been shown to modulate the levels of nitrites and nitrates, reducing the damage caused by nitric oxide metabolites following ischemic injury. Antioxidants such as vitamins A, C, and E failed to exhibit beneficial effects in metabolic diseases and aging, suggesting more potent and targeted antioxidant approaches [38]. The omega-5 NanoPSO may act through multiple pathways, including upregulating endogenous antioxidant defenses and inhibiting pro-inflammatory signaling cascades triggered by reactive nitrogen species [15]. In this way, we evaluated the superoxide dismutase enzyme, a key player in the regulation of nitric oxide signaling and antioxidant enzyme activity.

Omega-5 NanoPSO showed an increase in superoxide dismutase and could help restore the antioxidant defense system following ischemic injury. The cerebral tissue is particularly susceptible to oxidative stress due to its low levels of antioxidant enzymes, such as superoxide dismutase and glutathione peroxidase, as well as its high content of polyunsaturated fatty acids that are vulnerable to lipid peroxidation [15]. Alternative treatment with omega-5 NanoPSO is an excellent alternative for promoting endogenous antioxidant function and generating a neuroprotective effect. Interestingly, we discussed the recovery process after brain ischemia differs between animals and humans, complicating the direct extrapolation of treatments since animal models often recover more quickly and effectively due to differences in brain structures, such as less reliance on the pyramidal system than humans [39]. Unlike human rehabilitation, animal recovery protocols involve higher levels of repetition and controlled environments, which must consider factors like tolerability and variability in treatment responses. Hence, we need to perform more tests.

## 5. Conclusions

The role of omega-5 NanoPSO in brain ischemia is novel, facilitating the displacement rate and improving the working memory. We found the pivotal role of omega-5 NanoPSO in improving displacement and working memory associated with impairment after ischemic stroke. In addition, the treatment with omega-5 NanoPSO regulated the extravasation and reduced the astrogliosis phenomena of the inflammatory process, leading to increased damage. The survival neuronal cells in CA1 are crucial for establishing memory and locomotion, so the treatment with omega-5 NanoPSO was essential to the maintenance of neurons in the hippocampus. In the case of antioxidants, omega-5 NanoPSO was crucial in reducing the mechanisms of oxidative stress as such TAC, MDA, nitrites/nitrates, and increased the superoxide dismutase enzyme, which shows that it helps the body’s antioxidant systems. Interestingly, the group that showed noticeable changes was the group that received pre-treatment of omega-5 NanoPSO, which implies the preventive role of different nervous system pathologies.

## Data Availability

The original contributions presented in the study are included in the article/Supplementary Materials; further inquiries can be directed to the corresponding author.

## Supplementary Materials

The following supporting information can be downloaded at: https://www.mdpi.com/article/10.3390/antiox13111353/s1. Figure S1: Vertical and horizontal displacement in metatarsus on the third and seventh days after ischemia in rats; Figure S2: Vertical and horizontal displacement in the ankle on the third and seventh days after ischemia in rats; Figure S3: Vertical and horizontal displacement in the knee on the third and seventh days after the injury in rats; Table S1: Total differences in vertical and horizontal displacement (%) analyzing the control, brain ischemia injury, and omega-5 NanoPSO treatment groups.
