# Supplementary material for: Efficacy of Omega-5 NanoPSO Treatment in the Hippocampus, Through Antioxidant Mechanisms, After an Ischemia/Reperfusion Injury, in Murine Model
Source: Antioxidants (Basel). 2024 Nov 5;13(11):1353. doi: 10.3390/antiox13111353 (PMC11591363; doi:10.3390/antiox13111353)
Supplement: Supplementary file 1 [file antioxidants-13-01353-s001.zip › antioxidants-3265251-supplementary.pdf]

1. **Vertical and Horizontal displacement in metatarsus on the third and seventh days after ischemia in rats.**

We averaged the vector Y and X components at each end of the normalized displacement curves for each group. We compared every step curve of an animal three days post-injury to the curves of a different animal seven days post-injury. Thus, we calculated differences in the displacement curves between every ischemia animal to determine the differences between days. We compared this pattern comparison analysis using locally designed MATLAB 2023B Software.

To determine the changes in vertical and horizontal displacement of adult male rats after an ischemia injury, differences in the displacement of the metatarsal joint (Figure S1A, B, C, and D) of the hindlimb were analyzed. The difference in the vertical displacement of the left metatarsus of the 3-dpi group (black line) versus the 7-dpi group (red line) was 50% (A), and the right metatarsus was 33% (B). The difference in the Horizontal displacement of the left metatarsus of the 3-dpi group versus the 7-dpi group was 63% (C), and the right metatarsus was 14% (D). Asterisks above zero (*) show the points where the step cycle is different with statistical significance (P ≤ 0.05). The annotation at the bottom of each graph shows the total percentage of the statistically different cycle.

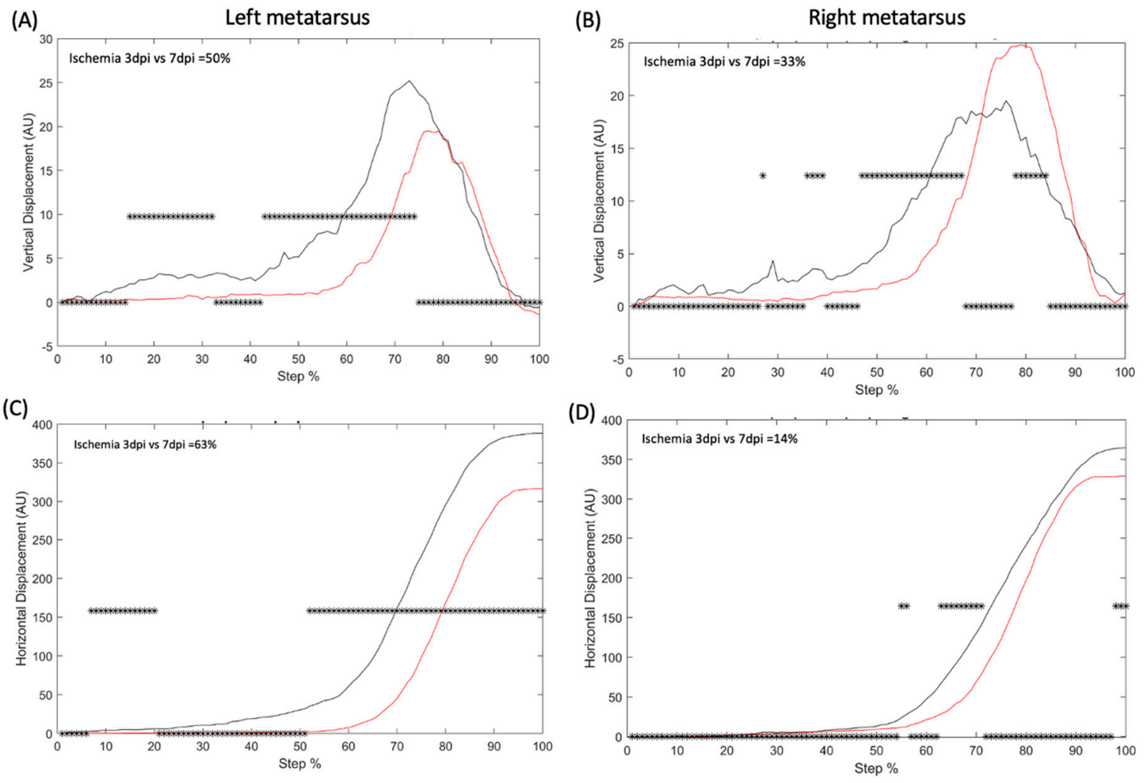

Figure S1. Graphs illustrate the metatarsus vertical (VD) and horizontal (HD) displacement of the injury group at 3 days versus 7 days in the left and right metatarsus joints, respectively. The asterisks illustrate the bins with a statistical difference (*P ≤ 0.05). The percent of change is expressed above the graphs. The step cycle was divided into 100 bins (cycle percentage). The cross above Zero indicates bins with a significant statistical difference. The red and black lines illustrate the ischemia at 3 and 7 days, respectively.

2. **Vertical and Horizontal displacement in the ankle on the third and seventh days after ischemia in rats.**

We analyzed the changes in vertical and horizontal displacement after an ischemia injury, as well as differences in the displacement of the ankle joint (Figure S2A, B, C, and D) of the hindlimb. The difference in the vertical displacement of the left ankle of the 3-dpi group versus the 7-dpi group was 29% (A), and the right metatarsus was 8% (B). The difference in the Horizontal displacement of the left metatarsus of the 3-dpi group versus the 7-dpi group was 94% (C), and the right metatarsus was 21% (D). Asterisks above zero (*) show the points where the step cycle is different with statistical significance (P ≤ 0.05). The annotation at the bottom of each graph shows the total percentage of the statistically different cycle.

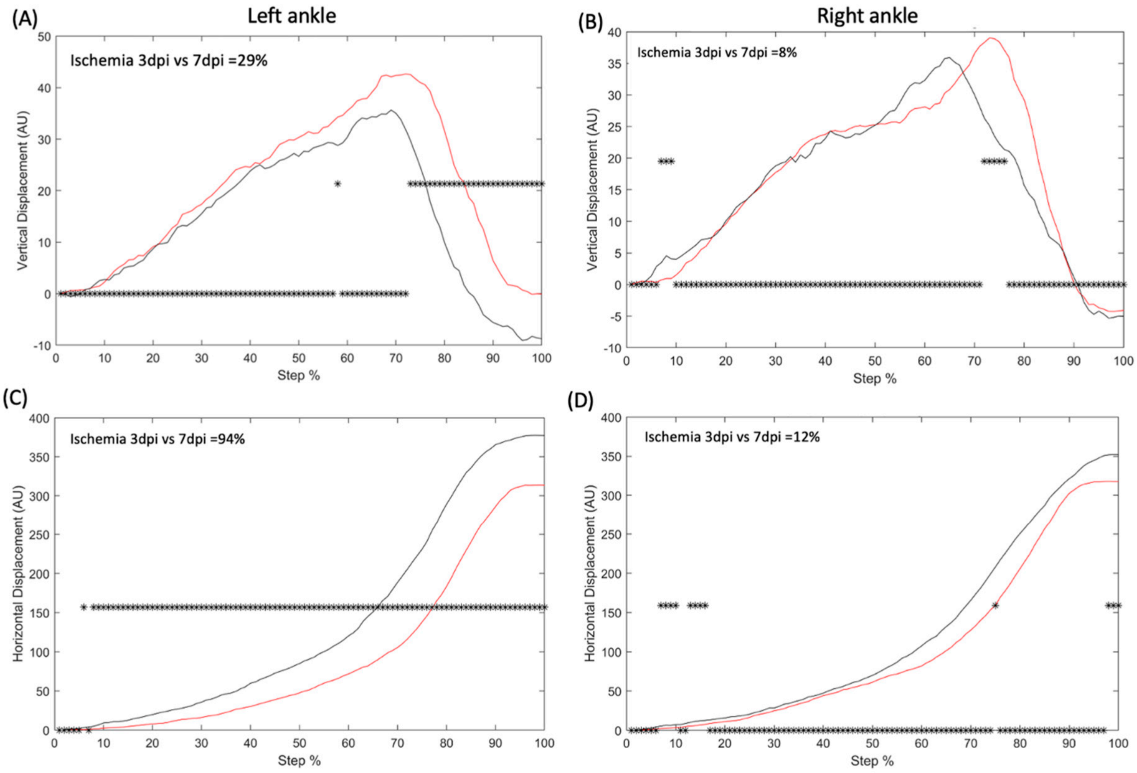

Figure S2. Graphs illustrate the metatarsus vertical (VD) and horizontal (HD) displacement of the injury group at 3 days versus 7 days in left and right ankle joints, respectively. The asterisks illustrate the bins with a statistical difference (*P ≤ 0.05). The percent of change is expressed below the graphs. The percent of change is expressed above the graphs. The step cycle was divided into 100 bins (cycle percentage). The cross above Zero indicates bins with a significant statistical difference. The red and black lines illustrate the ischemia at 3 and 7 days, respectively.

3. **Vertical and Horizontal displacement in the knee on the third and seventh days after the injury in rats.**
   We analyzed the changes in vertical and horizontal displacement after an ischemia injury, as well as differences in the displacement of the knee joint (Figure S3A, B, C, and D) of the hindlimb. The difference in the vertical displacement of the left ankle of the 3-dpi group versus the 7-dpi group was 10% (A), and the right metatarsus was 1% (B). The difference in the Horizontal displacement of the left metatarsus of the 3-dpi group versus the 7-dpi group was 87% (C), and the right metatarsus was not different (D). Asterisks above zero (*) show the points where the step cycle is different with statistical significance (P ≤ 0.05). The

annotation at the bottom of each graph shows the total percentage of the statistically different cycle.

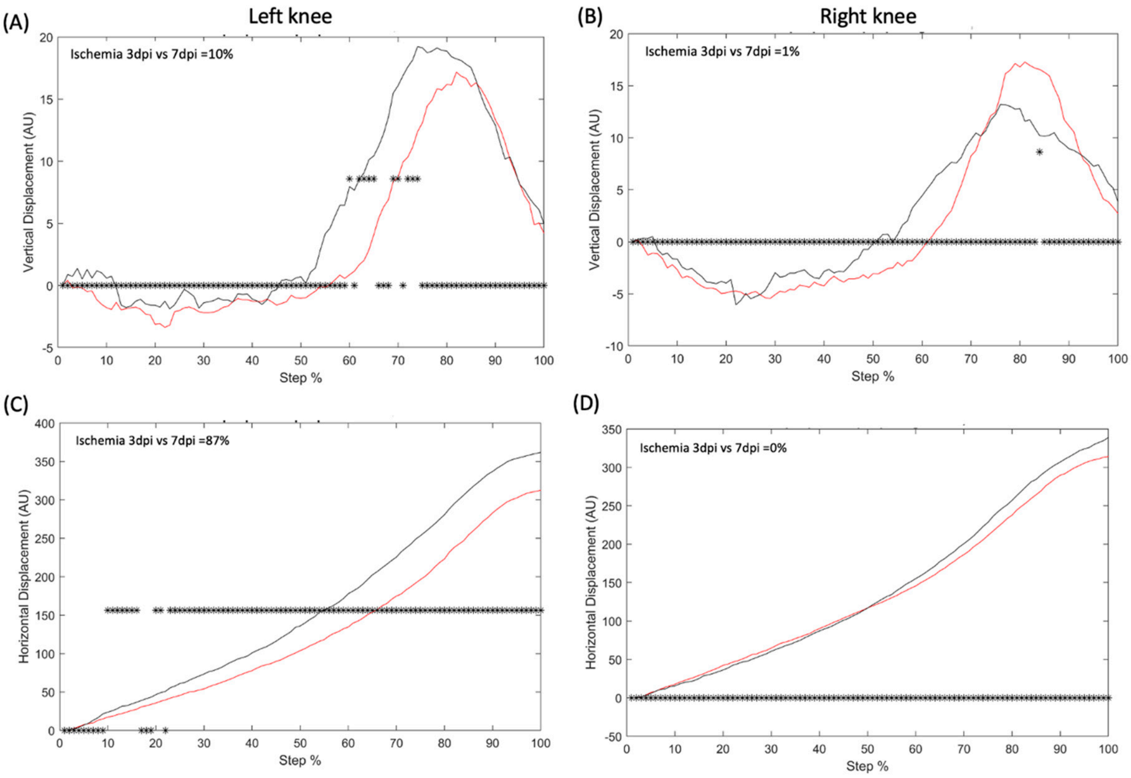

Figure S3. Graphs illustrate the metatarsus vertical (VD) and horizontal (HD) displacement of the injury group at 3 days versus 7 days in left and right knee joints, respectively. The asterisks illustrate the bins with a statistical difference (*P ≤ 0.05). The percent of change is expressed below the graphs. The percent of change is expressed above the graphs. The step cycle was divided into 100 bins (cycle percentage). The cross above Zero indicates bins with a significant statistical difference. The red and black lines illustrate the ischemia at 3 and 7 days, respectively.

4. **Total differences in vertical and horizontal displacement (%) analyzing the control, brain ischemia injury, and Omega 5 NanoPSO treatment groups.**

To have a broader overview of the changes in the kinematics of gait locomotion after brain ischemia injury and the effects of the post and pre-treatments with Omega 5 NanoPSO, a data concentrate corresponding to the changes in the Vertical displacement (VD) and Horizontal displacement that occurred between the experimental groups on both the left and right sides (Table 1). It is important to highlight the VD and HD that had minimal significant differences (0 or 1% of the step cycle) whether on the left or right side. These comparisons between groups were either at 3- or 7-days

post-injury, with minimal differences compared to the control group and pre and post-treatment with Omega 5 NanoPSO.

Table S1. Total Comparison (%) of brain ischemia to 3 and 7 days post-injury versus Omega 5 NanoPSO post and pre-treatment.

**Study groups**	**Metatarsus**				**Ankle**				**Knee**			
	**Left**	**Right**	**Left**	**Right**	**Left**	**Right**	**Left**	**Right**	**Left**	**Right**	**Left**	**Right**
	**VD %**		**HD %**		**VD %**		**HD %**		**VD %**		**HD %**	
**Control vs Ischemia 3dpi**	10	22	19	0	0	16	0	0	2	3	0	0
**Control vs Ischemia 7 dpi**	42	19	47	10	32	15	91	12	9	7	74	16
**Ischemia 3dpi vs Ischemia+NanoPSO**	**6**	12	**1**	10	**0**	4	0	11	19	3	**4**	14
**Ischemia 7dpi vs Ischemia+NanoPSO**	**10**	0	**31**	3	**0**	0	28	8	10	0	**67**	1
**Ischemia 3 dpi vs preNanoPSO+Ischemia**	**7**	25	**0**	0	**1**	14	**2**	42	0	6	**0**	0
**Ischemia 7dpi vs preNanoPSO+Ischemia**	**22**	8	**26**	12	**34**	27	**77**	14	2	0	**32**	26

dpi= days post-injury. The blue background represents the most specific changes when comparing the injury versus Omega 5 NanoPSO; Figures 2, 3 and 4, in the text

Table S1. Differences in Vertical and Horizontal displacement between the left and right side by group. The total differences with statistical significance in the step cycle per joint in the VD and HD between the left side and the right side of each experimental group are shown. A data concentrate corresponds to the changes in the Vertical (VD) and Horizontal displacement between the experimental groups on both the left and right hindlimbs.
